# Assessing antibiotic prescribing in nurse practitioners: Applied cognitive task analysis

**DOI:** 10.1016/j.ijnsa.2022.100101

**Published:** 2022-09-25

**Authors:** Nataly Martini, Je Wei Choong, Paula Donamae Dela Cruz, Helen Lau, Hanna Lim, Roger Liu, Anecita Gigi Lim, Dianne Marshall

**Affiliations:** a School of Pharmacy, Faculty of Medical and Health Sciences, The University of Auckland, Private Bag 92019, Auckland 1142, New Zealand; b School of Nursing, Faculty of Medical and Health Sciences, School of Pharmacy, The University of Auckland, Private Bag 92019, Auckland 1142, New Zealand

**Keywords:** Nurse practitioners, Drug prescriptions, Anti-bacterial agents, Inappropriate Prescribing, Antimicrobial Stewardship

## Abstract

**Background:**

Prescribing antibiotics is a demanding and complex task where decision-making skills are of critical importance to minimize the risk of antimicrobial resistance. Despite its importance, little is known about the decision-making skills and cognitive strategies new Nurse Practitioners (NPs) use when prescribing antibiotics.

**Objective:**

To identify the cognitive demands of antibiotic prescribing complexity and to explore the cognitive strategies that new NPs in New Zealand use when prescribing antibiotics.

**Design:**

A qualitative approach using Applied Cognitive Task Analysis (ACTA) methodology.

**Participants:**

A purposive sample was recruited consisting of five NPs who had been registered within the last five years and were prescribing antibiotics as part of their scope of practice.

**Methods:**

In-depth face-to-face interviews consisting of a task diagram interview and a knowledge audit were conducted and analyzed following the ACTA protocol.

**Results:**

Four cognitive elements were identified from the data which showed the cognitive demands of prescribing antibiotics, and the cues and strategies NPs use for safe practice. These were: 1 prescribing in the face of uncertainty (complex patients and diagnostic uncertainty); 2 making clinical decisions with insufficient/poor guidance (lack of guidelines, conflicting information); 3 producing an individualized treatment plan in view of clinical and non-clinical patient factors (patient demand/expectation, inadequate patient education, risks versus benefits of antibiotic treatment); 4 ensuring treatment efficacy and continuity of care (ineffective treatment, patient care follow up).

**Conclusion:**

The ACTA framework has given insight into the current antibiotic prescribing practice of new NPs, identifying areas where professional development courses and treatment resources can be targeted to support antibiotic prescribing. NPs are likely to benefit from resources that are freely available and reflect national or local antimicrobial data. Further work is also warranted to determine whether targeted education resources and clinical pathways will help with diagnostic uncertainty, and how this could be embedded into existing curricula.

**What is already known about the topic?**

Antibiotics are amongst the most prescribed medicines by nurse practitioners.

Although antimicrobial stewardship is a consideration, diagnostic uncertainty and the patient's clinical condition play a greater influence on antibiotic prescribing by nurse practitioners.

Clinical decision-making is influenced by the nurse practitioner's experience, specialty, knowledge, confidence, and critical thinking disposition.

What this paper adds

The Applied Cognitive Task Analysis Framework is a novel approach to exploring the cognitive demands and strategies that influence NP antibiotic prescribing practice.

The greatest cognitive demand resulted when NPs were faced with prescribing decisions for complex and high-risk patients, where guidelines weren't readily available, with pressure from patients and superiors, and when balancing risks versus benefits for individualized patient care.

NPs prioritized antibiotics based on efficacy, dosing frequency, route of administration, and appropriateness for the patient even though antimicrobial stewardship was considered a high priority.

Alt-text: Unlabelled box

## 1 Introduction

Antimicrobial stewardship programs promote the judicious use of antimicrobials to reduce antimicrobial resistance (MacDougall and Polk, 2005). Inappropriate antibiotic prescribing is a major driver of antimicrobial resistance and is associated with higher mortality rates, extended hospital stays, and increased healthcare costs (Weddle et al., 2017; Thomas et al., 2014). In New Zealand hospitals, 36% of antibiotics were estimated to be prescribed inappropriately (De Almeida et al., 2018). Common reasons for inappropriate prescribing include prescribing broad-spectrum antibiotics, prescribing when not indicated, and incorrect dosing. Although general practitioners (GPs) remain the main prescribers of antibiotics in primary care, other health professionals with prescriptive authority are accountable for 8% of all community antibiotic prescriptions (Courtenay et al., 2019). A recent New Zealand study found antibiotics to be amongst the most prescribed medicines by nurse practitioners (NPs), with over 70% prescribing a broad-spectrum antibiotic (Poot et al., 2017).

The extension of prescribing rights to allied health professionals has been granted under a variety of regulatory and legislative models. In New Zealand, NPs were given prescribing rights in 2002, allowing them to prescribe medicines from a gazetted list of drugs (Lim et al., 2014). In 2014, legislation changes gave NPs full authorized prescribing rights that permitted independent prescribing of any medicine within a specific area of practice (Poot et al., 2019; Nurse practitioner 2020). This was revised by the Nursing Council of New Zealand (NCNZ) in 2017 to remove restrictions on specific areas of practice and broadened the scope of prescribing practice to include all medicines within their competence and clinical experience (Nurse practitioner 2020). To meet core competencies, NPs must demonstrate advanced diagnostic and therapeutic knowledge, and sound clinical reasoning skills to assess, diagnose, plan, implement and evaluate care (Nurse practitioners in New Zealand [Internet] 2020). Clinical reasoning draws on theoretical, evidence-based medicine and practice-based knowledge to guide and evaluate the best possible treatment regimens for patients and is essential when prescribing (Nurse practitioners in New Zealand [Internet] 2020).

Clinical reasoning and decision-making processes have been widely explored in NPs, demonstrating that NPs approach decision-making holistically (Burman et al., 2002), social and institutional factors play an important role in the decision-making process (Offredy, 1998), and with expertise, NPs use both the Information Processing Model and Hermeneutical Model (Ritter, 2003). However, as a relatively new group of prescribers, literature identifying the cognitive factors that influence NPs’ antibiotic prescribing practice is still largely lacking. Existing research has acknowledged numerous cognitive influences on NPs’ antibiotic prescribing behavior, of which diagnostic uncertainty and the clinical condition of the patient have been found to influence prescribing the most (Courtenay et al., 2019; Rowbotham et al., 2012; Ness et al., 2016). This can be attributable to fear of making the wrong clinical decision, especially in high-risk or vulnerable population groups (Courtenay et al., 2019; Rowbotham et al., 2012; Ness et al., 2016). Additionally, NPs perceive themselves to be open to scrutiny by medical prescribers and lacking in legal protection (Rowbotham et al., 2012). As such, NPs have been found to mostly rely on published guidelines when prescribing antibiotics (Courtenay et al., 2019; Rowbotham et al., 2012; Ness et al., 2016; Hannigan et al., 2012; Goolsby, 2007). Where these are not readily available, peer discussion was used to support decision-making (Rowbotham et al., 2012; Ness et al., 2016; Williams et al., 2018). Antibiotic prescribing decisions were further guided by patient requirements for an antibiotic (Hannigan et al., 2012), and the NPs experience and familiarity with the antibiotic (Sanchez et al., 2014). Non-medical factors such as antibiotic cost, the patient's race, and socioeconomic status also influenced antibiotic prescribing, but this was not consistently reported in the literature (Williams et al., 2018). Patient demand and expectations for an antibiotic (Courtenay et al., 2019; Rowbotham et al., 2012; Ness et al., 2016; Hannigan et al., 2012; Williams et al., 2018; Sanchez et al., 2014), and inconsistent approaches between prescribers, undermined NPs prescribing decisions (Rowbotham et al., 2012; Williams et al., 2018; Sanchez et al., 2014), often resulting in a patient being given a delayed prescription. This was seen as a way to compromise with patients who strongly believed they needed antibiotics, and delayed prescribing is well-supported for upper respiratory tract infections (Spurling et al., 2017). Although antimicrobial resistance was a prescribing consideration, it was not a priority, ranking eighth among eighteen factors when making antibiotic treatment decisions (Hannigan et al., 2012).

Given the need for NPs to lead antimicrobial stewardship, it is critical to understand the complex cognitive strategies employed by NPs when prescribing antibiotics. Literature shows us that clinical decision-making knowledge and skills are influenced by factors such as years of NP experience, specialty, foundational and professional knowledge, clinical confidence and critical thinking disposition, and are likely to be strengthened through reflective practice (Chen et al., 2016). Skillful practitioners are those with an awareness of their cognitive processes and are better able to assess their knowledge, are more strategic in their reasoning, and are better able to handle complexity (Ku and Ho, 2010). However, due to the implicit nature of clinical reasoning, it is often difficult for individuals to articulate, formalize and explain this knowledge (Gascoigne and Thornton, 2014; Marshall and Finlayson, 2022). Furthermore, expert practitioners often omit critical information as knowledge becomes automatic and bypasses conscious processes (Crandall et al., 2006). Consequently, the cognitive strategies that NPs use when prescribing are ill-defined (Elkhadragy et al., 2019). Even less is known about the cognitive impact on NPs as they transition from an expert nurse to a novice NP (Fitzpatrick and Gripshover, 2016). The cognitive effort required for novice/new NPs to select the most appropriate medication and write a prescription is extensive, with novices more reluctant to take risks in prescribing, and referring these decisions to experienced doctors (Lim et al., 2018). Development of expertise can take months or years (Lim et al., 2018).

This study aims to explore the cognitive demands that NPs new to prescribing experience, to reduce inappropriate prescribing and support antimicrobial stewardship initiatives. To meet this aim, Applied Cognitive Task Analysis (ACTA) was selected as a method. ACTA is a cognitive psychology technique that examines unconscious knowledge (Marshall and Finlayson, 2022) and provides an opportunity to examine the elements of prescribing complexity and identify the cognitive strategies that NPs use when prescribing antibiotics. It is expected that by identifying these factors, education could be better targeted at new NPs to enable safer and more effective antimicrobial prescribing.

## 2 Methods

### 2.1 Study design

Applied Cognitive Task Analysis (ACTA) methodology was used to identify strategies and cues that NPs use when prescribing antibiotics. ACTA has its roots in cognitive psychology and from a philosophical perspective is best aligned with naturalistic inquiry where researchers are concerned with interacting with individuals who have real-life experience and first-hand knowledge of the phenomenon of interest within its context. ACTA is unique in that it uses a wide range of knowledge-elicitation and -representation techniques that build on each other to investigate different aspects of cognition. It explicitly considers the cognitive processes associated with an activity and systematically analyses the decisions or choices individuals make when performing a task. Studies using cognitive task analysis (CTA) interview methods have shown better capture and accuracy of decisions and action steps about performing a task compared to free-recall interviews often used in qualitative approaches (Clark et al., 2012). Additionally, ACTA provides a toolkit of interview methods that enables the extraction of information about the cognitive demands required for a task (Crandall et al., 2006; Militello and Hutton, 1998). This method was selected as it can expose implicit knowledge that is often difficult to verbalize. It is known to elicit the cognitive skills that are essential for skilled performance and decision-making (Crandall et al., 2006) and has successfully been used to understand expertise in the health care domain (Marshall and Finlayson, 2022; Morozova et al., 2017; Marshall, 2022). Furthermore, unlike traditional CTA methods that are resource intensive, ACTA requires little training to implement as the toolkit provides comprehensive training material for researchers.

Two rounds of semi-structured interviews comprising a task diagram interview and a knowledge audit interview were utilized in accordance with the ACTA method to collect data. The knowledge audit interview was adapted to include a probe on antimicrobial stewardship to fit with the purposes of this qualitative study. Before initiating the interviews with the recruited participants, a pilot study with one NP was conducted.

### 2.2 Participant recruitment

Participants of this study were NPs who, at the time of the study, were practicing and prescribing antibiotics in NZ in an extensive scope of practice such as primary healthcare or emergency department, and who registered as an NP within the last five years (2013- 2018). Due to the limited number of NPs who were new prescribers, a purposive sampling method (Creswell, 2013) was considered the most appropriate technique to recruit participants. Participants’ level of practice and their willingness to participate determined their inclusion in the study. The number of participants chosen was in accordance with ACTA methodology (Militello and Hutton, 1998), which suggests three to five participants are usually sufficient to exhaust critical information about the cognitive skills required to perform a particular task safely.

Nurse Practitioners New Zealand (NPNZ) agreed to advertise the study to their members. They sent out a study invitation to potential participants via email with an attached participant information sheet and researcher contact details. This resulted in five volunteers agreeing to be interviewed.  The first five NPs who met the inclusion criteria were selected for the study. Participants gave written consent prior to data collection.

### 2.3 Data collection

Each interview was scheduled for 60 min and was conducted at the NP's workplace by two undergraduate final year Pharmacy student researchers. One student took the role of lead interviewer and asked questions developed from the ACTA toolkit; the supporting interviewer ensured that all questions were covered and prompted elaboration on certain points where appropriate. Students had undergone an intensive research course prior to conducting the interviews and were trained in the ACTA interview technique by supervisors familiar with the method. Students were also trained to build rapport with participants prior to study commencement. Interviews were audio-recorded with permission from the participants.

The ACTA interview is conducted in two parts. The first part, a task diagram, provides an overview of the major steps taken when performing a task. For the task diagram, each NP was asked to describe their clinical decision-making process when prescribing antibiotics for a patient by illustrating this with a three to six-step task diagram. From these steps, the NP was asked to identify which steps they believed required the most cognitive demand. The steps identified served to focus the second part of the interview, the knowledge audit.

The knowledge audit makes use of a series of six probes (with an option to include more) from the ACTA toolkit to elicit deeper information on decision-making processes and areas that require expertise (Militello and Hutton, 1998). In our study, we included optional questions on antimicrobial stewardship and anomalies that may arise when prescribing antibiotics for certain population groups. The probes identified the reason why the steps identified in the task diagram were cognitively demanding and elicited the cues and strategies NPs used to address cognitively demanding situations when prescribing antibiotics.

1 Example questions asked for each probe were:
  Big picture: Can you give an example of what is important about the big picture for antibiotic prescribing?
2 Noticing: Have you had an experience when managing an infection where you noticed things going on that others did not?
3 Job smarts: When you prescribe antibiotics, have you found ways of working smart to accomplish more with less that you have found especially useful?
4 Opportunities/ improvising: Think of a time when you did not have the resources to guide antibiotic selection or prescribing. How did you manage the situation?
5 Past and future: Can you describe a situation you find/found challenging when seeing patients with different kinds of infections in clinical practice?
6 Self-monitoring: Can you think of a time when you realized that you would need to change the way you were prescribing antibiotics to optimize patient outcomes?
7 Antimicrobial stewardship: What is your understanding of antibiotic resistance?
8 Anomalies: Can you describe a time when a patient did not respond to the antibiotic that you gave them? What factors would, or did, you think about? What are the characteristics of the population you see that require you to prescribe an antibiotic?

### 2.4 Data analysis

ACTA analysis consists of three stages resulting from the interviews: (1) a task diagram flowchart highlighting the cognitively demanding step(s) in antibiotic prescribing, (2) a knowledge audit table developed from probes to identify specific cognitive information relating to the cognitively demanding elements of prescribing antibiotics, and (3) a cognitive demands table consolidating data gathered from the task diagram and knowledge audit to present the overall findings of the ACTA.

Upon completion of the interviews, a master task diagram was developed by combining common elements from the five individual task diagrams to provide a summary of the findings. Each knowledge audit interview transcript was read to identify the key points from the interview according to the relevant probes. For each knowledge audit an individual knowledge audit table was constructed considering the (1) NP's aspects of expertise, (2) cues and strategies used in dealing with the cognitively demanding situation, and (3) reason why the situation would be difficult/demanding. The individual knowledge audit tables were then compared and combined to construct a master knowledge audit table. Responses to probes that were ambiguous were discussed as a research team, and through discussion, an agreement was reached on where these should be presented in the table.

The master task diagram and master knowledge audit table were combined into a cognitive demands table (Table 2) to enable the tacit cognitive demands of prescribing antibiotics to be made visible. This process involved systematically working through the findings of all interviews for common themes as well as conflicting information. Information relevant to the aims of the study was then categorized according to the following categories: difficult cognitive demands, why they are difficult, and cues and strategies NPs used to address the difficulty.  For each component, each researcher analyzed the data individually before meeting collectively to compare and identify any discrepancies. These were then discussed and resolved to ensure that the results were robust and consistent. This was done to minimize any potential bias and consider the interpretations and perspectives of all researchers.

To ensure the rigor of the study findings, the researchers were guided by Lincoln and Guba's (Lincoln and Guba, 1985) four criteria for establishing trustworthiness: credibility, transferability, dependability, and confirmability. To increase credibility and reduce the possibility of biased decisions, each researcher analyzed the data individually prior to meeting collectively to compare for agreement. Transferability was supported by the use of thick description of the interview data to support the findings. Participants’ direct quotes from the recorded interviews were used to illustrate key points of analysis to determine the applicability of findings to other clinical settings and populations. To address the issue of dependability, detailed accounts of the research design, data collection methods and data analysis have been provided to enable replication of the study. To reduce investigator bias and to ensure the findings resulted from the NPs experiences and not the perspectives of the researchers, confirmability was supported by encouraging the researchers to use reflective diaries to explore thoughts and feelings that could impact on data collection and interpretation.

### 2.5 Ethical considerations

Ethics approval for this research was obtained from the University of Auckland Human Participants Ethics Committee (Reference number 021,338). Participants were provided with a participant information sheet explaining the goals of the research, the reasons for the research, and who the researchers were. Informed consent was obtained from all NPs prior to data collection, and all participants were free to withdraw at any stage without consequence. Data were stored in a password-protected file on a secure university server.

## 3 Results

### 3.1 Participant demographics

Of the five participants in this study, all were registered as an NP for two years or less (range 8 months – 2 years); three worked in primary healthcare (Table 1).

**Table 1:** Characteristics of study participants.

Participants	Gender	Years registered as NP	Practice setting
NP01	Female	2 years	Primary Healthcare
NP02	Female	2 years	Primary Healthcare
NP03	Female	8 months	Secondary Healthcare
NP04	Female	1 year	Secondary Healthcare
NP05	Female	9 months	Primary Healthcare

### 3.2 Master task diagram

**Fig. 1:**
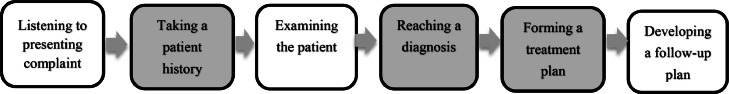
Master task diagram (shaded in gray are steps that required the most cognitive demand).

The antibiotic prescribing steps by NPs were combined and compiled into the master task diagram (Fig. 1). Six task steps were determined as critical components by the NPs when prescribing antibiotics. The most cognitively demanding steps for all participants were taking a patient history, reaching a diagnosis, and formulating a treatment plan.

Taking a patient history required comprehensive information on medical history and presenting complaint to aid diagnosis and a treatment plan. Cognitive challenges arose where histories were complex, and patients did not provide sufficient information.

“*Some patient's history is very difficult. Some patients don't tell you initially. They just come and sit in front of you and start coughing. That's all. Nothing else.*” (NP05)

In these instances, NPs further questioned patients to prompt elaboration on their complaint, and considered other aspects such as social history to aid decision-making.

Reaching a diagnosis was found demanding due to the shift in the scope of practice and responsibilities from a registered nurse to an NP.

*“As a registered nurse you don't diagnose. And that's kind of drummed into you. And so, then that change in practice as a nurse practitioner. That's quite a different kind of culture and expectation.” (NP02)*

Furthermore, NPs did not always think about differential diagnoses and found this a big challenge while increasing their scope of practice. Likewise, cognitive demand arose when the clinical assessment and examination findings deviated from the suspected diagnosis.

“*The whole history sounded like they'd broken their hand, but they didn't have much bony tenderness, it just sounded like a trauma. I sent them off for X-rays, then nothing was in the X-ray. It just looked really bizarre, it looked like a swollen hand but what I hadn't thought about was that he had track marks further up his arm, and I just kept thinking about what else could it be. And it was only when I got a colleague in and they said, ‘Oh are they track marks up here and running downwards?’ They'd been injecting IV.*” (NP03)

NPs made use of clinical guidelines to aid diagnosis and continuing education to build on diagnostic skills, recognizing complex diagnoses as an ongoing challenge.

Formulating a treatment plan that was appropriate for each patient was cognitively demanding. This was made more demanding by the patients’ expectations, demands and circumstances. Education and self-care advice was not always readily accepted by the patient if they desired antibiotics.

“*The toughest task, is where I have a patient who believes 'if I do not have antibiotics I won't get better.*' *If the patient is really sure they need antibiotics and I'm really sure they don't, and we can't find a meeting place in the middle. That's tough.*” (NP01)

Although complex medical and social history were considered when selecting an antibiotic, deciding on the treatment plan was guided by the ability, and willingness, of the patient to take the medicine.

“*Deciding which would be the best* [antibiotic] *to use for that patient, and the best dosing. Do they take it once a day or twice a day? Is it too big for them to swallow? How much does it cost? So, it's thinking through all of those processes. You can't make the same plan for every patient. Just making the right choice for what's best for that patient - that's the tricky bit*” (NP04)

Decisions made not to treat with antibiotics were sometimes overruled by other clinicians.

*“As soon as the GPs get the X-ray result, they'll put the patient on antibiotics. So, even though I might make a decision not to treat, I get overruled. Someone else will treat them.”*

### 3.3 Master knowledge audit

A master knowledge audit table was compiled from individual knowledge audit interviews (supplementary file). Further insights were provided through specific quotes from the interviews.

#### 3.3.1 Big picture

The antibiotic indication was determined by conducting a thorough patient history and physical examination. NPs considered the site and severity of the infection, diagnosis, likely causative organism, and antibiotic appropriateness when making clinical decisions. Patient's symptoms, previous treatment and adherence to the treatment plan were also considered, as well as the population risk factors and antibiotic resistance patterns. The clinical indication for an antibiotic was a primary consideration when evaluating a patient with an infection.

“…*the first I think about is, are antibiotics appropriate in this situation? I mean… there's no point thinking beyond that to what type and what's it for, or anything like that until, you know, first question, is that a consideration? Because if it isn't then I'd look up other options.” (NP01)*

Patients with complex comorbidities and allergies were considered more demanding to manage because treatment options were limited, affecting decisions. High-risk patients were more likely to be prescribed antibiotics.

*“…maybe antibiotics might be considered for the chest but then you look and they also have an ear infection. Then you think well, normally, you wouldn't treat that with an antibiotic either, but they are high risk for whatever reason, what's something that will cover both infections?” (NP01)*

Similarly, treatment for pediatric patients was demanding due to parents’ expectations for treatment. In these situations, NPs needed to weigh the risks and benefits of not prescribing antibiotics.

*“If you think that the patient won't bring the child back, then we have to give antibiotics that time. So, depends on the parents and their ability to look after themselves, I find that really affects the prescription.” (NP04)*

Pressure to prescribe antibiotics from superiors undermined decision-making.

#### 3.3.2 Noticing

NPs used clinical observations, diagnostic tests, and comprehensive medical history to assess the severity of the infection, and integrated their expert diagnostic skills with the clinical presentation when prescribing antibiotics. Where patients were at high risk, antibiotics were not delayed.

“*Like severe cellulitis, it's gonna be IV antibiotics. But yeah, there are certain presentations that you go, that needs treating now or it's going to hospital. So yes, definitely there are some situations where you're still gonna go through it but it takes* [snaps finger] *this long instead…*” *(NP01)*

Novice prescribers were thought to experience greater cognitive demand when the diagnosis was uncertain. In these beginners, pattern recognition was considered to be unreliable.

*“Quite often* [novice nurse practitioners] *see a red eye and think 'if they need antibiotics, they need antibiotics' so yeah that's a common one.” (NP02)*

#### 3.3.3 Job smarts

With clinical experience, NPs refined their diagnostic and patient management skills. Over time, NPs relied more on pattern recognition when diagnosing and making decisions on the need for antibiotics.

“*It's not like cutting corners, it's about learning the most efficient way of doing things. So, in my examination now, I probably don't do as extensive an examination as I used to, because now that I've done lots of those, I know these are the things I need to focus on, and if any of these come up, then I'll look further. But if none of those come up, I can be fairly confident that there's nothing else in there.” (NP01)*

NPs acknowledged the tensions that exist between time management and efficiency and the risk associated with missing something important. Working in partnership with their patients to ensure that there was mutual understanding about the treatment plan and consultation outcome was considered crucial. Good rapport with patients and working in a multidisciplinary team helped to manage patient pressure for antibiotics, improve adherence, and facilitated the delivery of a comprehensive service.

*“If you don't give patients good advice that's when they'll come back or won't come back when they should come back, or they won't go and see their GP…Yes,* [it] *definitely* [takes time], *but it is worth it…” (NP03)*

#### 3.3.4 Opportunities/Improvising

NPs took advantage of opportunities to extend their clinical knowledge and scope of practice and keep up to date with antimicrobial guidelines through self-study. At times, NPs needed to work around the guidelines by assessing the patient's response to treatment and re-evaluating treatment.

*“If the patient's not responding to the first treatment, then we need to think about a different cause for the problem. So, we had to reassess again, we had to think more broadly.”(NP05)*

Where information from guidelines was conflicting or lacking, NPs sought advice from other clinicians. In some cases, information required specialist interpretation.

*“…when you put in the interaction, it comes up with red, yellow, green, as to how severe it is. Sometimes it is the hypothetical reaction. And then you're like should I, shouldn't I? If it's a kidney problem, say if it's something that interacts with the kidneys but it's not a bad interaction, it's only hypothetical, you can prescribe it for a few days.” (NP03)*

One NP had a good relationship with a pharmacist who would pre-package a supply of antibiotics through a Medical Practitioner's Supply Order (MPSO), which would be supplied to the patient if the NP was concerned about their ability to fill the prescription. Due to restrictions on medicines that can be supplied on MPSO, the prescribed antibiotics were not necessarily first-line as per clinical guidelines.

#### 3.3.5 Past and future

Early treatment interventions could be implemented when the NP was able to determine how the situation developed and foresee its progress. In these circumstances, pattern recognition was used to guide antibiotic prescribing.

“*This is probably the only one where you instantly they walk into the room and they're limping and pull their trouser and you see circumferential erythema and you go okay, bam. It takes two seconds.” (NP01)*

Diagnostic uncertainty, and predicting risks of non-prescribing, posed ongoing challenges while NPs increased their scope of practice. In cases of uncertainty, NPs would either prescribe antibiotics or provide a back-pocket prescription (also referred to as delayed antibiotic prescribing).

*“If it is an infection, how risky it is for the patient if I don't give antibiotics now? So, that risk and antibiotic stewardship needs to be balanced. So, sometimes it's really hard…a very fragile patient, if we miss an infection, if that significantly impacts that patient's life, then we need to give antibiotics. Yeah, I will give antibiotics. At that time, I will consider the patient's expectation as well.” (NP05)*

#### 3.3.6 Self-monitoring

NPs reflected on their competency by comparing their previous practice to the currently recommended standards. This enabled them to continue to improve their prescribing practice.

*“…as a clinician I think we're always looking at our practice; hopefully; always looking at our practice and going, 'Okay, what needs honing, what needs updating,' so yes, but not in a way of “I got that really wrong.”(NP01)*

NPs expressed more confidence in practicing independently compared to when they initially started in their role.

*“With all the new information that's come out the past couple of years, if I'd been an NP earlier, I will think, maybe I would have prescribed them more, but now with everything sort of, come out about antibiotics, I'm more cautious. So, not that I'd prescribed them before, but I keep that in mind with my practice.” (NP04)*

Although NPs invested in antimicrobial resources, this was demanding for many who didn't have easy access or found these too costly without workplace support.

#### 3.3.7 Antimicrobial stewardship

NPs practiced antimicrobial stewardship to varying degrees, which was heavily influenced by the population they cared for in practice.

“*There's a lot of information out there now that you don't have to give* [prophylactic antibiotics] *for everything. So, if* [patients] *are fit and healthy and don't have any lowered immunity, for some of the things we used to give antibiotics out prophylactically, we stopped doing that now.” (NP03)*

Difficulties arose in managing patient expectations of treatment and practicing antimicrobial stewardship. Where patients expected antibiotic treatment, NPs attempted education as a strategy but also gave back pocket prescriptions. This was also a strategy followed if it was difficult or unlikely to follow up on the patient's progress.

“*The patient expectation is very important, so we have to act according to the patient expectation sometimes. When we come back to antibiotics, if the patient wants some antibiotic, that's a different case. We can help educate the patient, maybe we can give back-pocket antibiotic prescription.” (NP05)*

Some NPs were influenced by patient pressure to prescribe antibiotics. Managing expectations was considered important to minimize the risk of complaints if their management plan wasn't effective. Others faced pressure from their seniors, who themselves prescribed antibiotics for viral infections.

*“Probably that we are giving them out for viral infections way too many times. And that does become an issue sometimes, when you work with someone that thinks you should be giving them out, when you shouldn't be…but they're actually your boss… when you go talk to the senior consultant of the day and they recommend something else then you're obliged to take that information. That can be a little bit of an issue.” (NP03)*

#### 3.3.8 Anomalies

NPs acknowledged that their diverse patient population with different cultural backgrounds influenced their clinical decisions. Patients from diverse cultural backgrounds had different expectations for antibiotics, and education initiatives for those with limited language comprehension was demanding.

“*So firstly, if you're thinking about the broader health impacts, the home environment might be less healthy,* [Māori and Pacific populations] *may have overcrowding, they may have cold homes, they may have damp homes. Risk of spread. We, routinely for our children, we will do a throat swab and start them straight away on antibiotics for rheumatic fever. Whereas at the other practice we hardly ever swabbed them and definitely didn't give antibiotics generally. So, where we're practicing and that demographic and what their health risks are does make a difference.” (NP01)*

### 3.4 Cognitive demands table

Data from the master task diagram and the master knowledge audit tables were synthesized and presented as a cognitive demands table (CDT) (Table 2). The CDT summarises information about the cognitive demands placed on NPs when prescribing antibiotics. Overall, four elements showing cognitive demand were extracted from the dataset: (MacDougall and Polk, 2005) prescribing in the face of uncertainty; (Weddle et al., 2017) making clinical decisions with insufficient/poor guidance; (Thomas et al., 2014) producing an individualized treatment plan in view of clinical and non-clinical patient factors; (De Almeida et al., 2018) ensuring treatment efficacy and continuity of care. The CDT provides a complete picture of the cognitive challenges for NPs during prescribing of antibiotics. It presents the cognitive demand underpinning each cognitive element and the cues and strategies used by NPs to support proficient decision-making for prescribing antibiotics.

**Table 2:** Cognitive demands table.

Difficult cognitive element	Why difficult?	Cues and strategies used by NPs
1) Prescribing in the face of uncertainty.	Complex patients. Not knowing how a patient's medical and social history influences their presenting complaint. Differential diagnosis with rare or unfamiliar presentations.	Collect and integrate pertinent patient considerations in clinical decision-making, including: Severity of health status Level of health literacy Comorbidities Medical history Medications Socioeconomic status Sensitivities Allergies Conduct relevant clinical examination and initial assessments, like bacterial sensitivity swabs, to obtain objective results for diagnosis. Use knowledge and clinical experience to differentiate conditions and recognize disease patterns.
1) Making clinical decisions with insufficient/poor guidance.	Lack of New Zealand-specific studies on infectious disease treatment. Conflicting information from guidelines, recent research and/or colleagues/superiors.	Use available resources such as: International journals Guidelines - general (e.g. Best Practice Advocacy centre NZ) and specialized (e.g. Heart Foundation) New Zealand Formulary Goodfellow Gems* Health Pathways Invest time and finances to access subscription resources to improve competency. Seek support and collaboration with other clinicians.
1) Producing an individualized treatment plan in view of clinical and non-clinical patient factors.	Patient demand/expectations from personal perspectives and/or experience of antibiotic prescribing in other countries. Inadequate patient education or self-management. Considering risks and benefits of treating with antibiotics and selecting the appropriate antibiotic course.	Don't prescribe antibiotics unless necessary or provide a ‘back-pocket’(delayed prescribing) antibiotic prescription to patients whose condition does not improve after expected resolution. Provide patient education focussing on the need for antibiotics. Key information provided in writing for the patient's convenience. Provide self-care advice when antibiotics are not prescribed. Select antibiotic regimen based on evidence-based efficacy, dosing frequency, route of administration, and appropriateness for the patient.
1) Ensuring treatment efficacy and continuity of care.	Patient's condition may not be improving due to non-adherence or ineffective treatment. Workplace environment – difficulty in following up on patient care through primary and secondary care systems.	Reassess the patient's condition through additional tests (swabs, susceptibility testing). Reassess treatment regimen, adherence, and antibiotic resistance patterns. Consult with colleagues to consider other possible causes or treatment. Ensure continuity of care through collaboration between secondary care providers, the patient and health professionals in primary care.

⁎ Goodfellow Gems, produced by the Goodfellow Unit, are brief 100–150 educational reports on the latest research. These are not intended as clinical advice.

## 4 Discussion

To the best of our knowledge, this is the first study to use the ACTA framework to examine the cognitive strategies and approaches that NPs use to guide their antibiotic prescribing practice. This approach has revealed four main cognitive demands that NPs face when prescribing antibiotics, namely: (1) prescribing in the face of uncertainty; (2) making clinical decisions with insufficient/poor guidance; (3) producing an individualized treatment plan in view of patient clinical and non-clinical factors; (4) ensuring treatment efficacy and continuity of care. These findings are consistent with the existing literature on NP antibiotic prescribing practices and demonstrate that ACTA is effective in identifying cognitive demands associated with antibiotic prescribing.

NPs in this study found diagnostic uncertainty to be cognitively demanding. When presented with complex or unfamiliar presentations, NPs conducted further investigations to recognize disease patterns or sought advice from their colleagues. As NPs gained experience, they increasingly relied on pattern recognition to identify the cause of an infection and predict its progress, refining their diagnostic and patient management skills, and conducting examinations faster. Decision-making in NPs involves both analytical and intuitive cognitive approaches and is dependent on the NP's specialty, professional knowledge, skills, reflective and critical thinking disposition, as well as the complexity and uncertainty of the clinical problem (Chen et al., 2016). Pattern recognition is believed to be an expert analytical skill that develops with experience and knowledge and leads to an ability to discern which aspects of a situation are more important (Rogers and Steinke, 2022). At the beginner stage, the practitioner may have difficulty in identifying what is important or relevant in a complex situation, but it is expected that as competence develops, there is greater confidence and less reliance on peers and other healthcare professionals (Rees and Hays, 1996; Benner and Tanner, 1987). Expert nurses require fewer critical cues, and after two years of clinical experience, NPs’ consultation techniques are found comparable with those of medical doctors (Thompson et al., 2017).

In our study, antibiotic prescribing was heavily influenced by the population NPs cared for in practice. High-risk patients, who faced poorer health outcomes if an infection was untreated or missed, were more likely to be prescribed antibiotics without delay. In New Zealand, prescribers are encouraged not to delay treatment in Māori and Pacific populations as it is well recognized that rheumatic fever and skin sepsis are disproportionately high (Duffy et al., 2018) with antibiotics under-prescribed relative to disease burden (Metcalfe et al., 2019). Studies have shown that the possibility of missing an infection (Rowbotham et al., 2012; Ness et al., 2016; Roumie et al., 2005; Ladd, 2005) or treating vulnerable populations such as children and immunocompromised patients was more likely to result in a prescribed antibiotic, even if the diagnosis was unclear (Goolsby, 2007). NPs feel more accountable when prescribing for vulnerable groups and tend to prescribe broad-spectrum antibiotics to ensure spectrum coverage (E. Ladd, 2005; Dempsey et al., 12; Wright et al., 2019). This is especially true for patient groups living with the lowest median household income who are at risk of worse health outcomes owing to poor access to healthcare (Ladd, 2005a; Ference et al., 2016) and greater susceptibility to post-infection complications (Ladd, 2005a). As NPs gain experience and clinical knowledge in specific population groups, they are better able to develop a “matrix of comparisons” and expectations taking subtle differences and variations for that population group into account. This can help to predict clinical outcomes and allows for expert diagnostic and intervention skills (Benner et al., 2008).

Once established that the patient required an antibiotic, prescribing was directed by local antimicrobial guidelines, accessibility to and familiarity with the antibiotic. Cognitive difficulties arose when guidelines were not readily available or uncomprehensive, or when information was conflicting and NPs were unsure about local susceptibility patterns. In these situations, guidance was sought from peers, or patients were referred to the hospital for further investigation and/or treatment. Reliance on guidelines is extensively reported in the literature (Courtenay et al., 2019; Rowbotham et al., 2012; Ness et al., 2016; Hannigan et al., 2012; Goolsby, 2007). Antibiotic prescribing that is not concordant with guidelines can result in overprescribing and can increase the risk for antimicrobial resistance. However, it is acknowledged that in many clinical situations, there are no clear guidelines and/or clinical trials to guide decision-making (Benner et al., 2008). In such cases, the NP must be able to draw on their knowledge and clinical experience to ascertain what evidence is relevant to their patient's situation, and how that differs from the general scientific understanding, recognizing that the process is complex and evolving as new evidence comes to light (Benner et al., 2008). Studies have shown that deficiencies in undergraduate knowledge of bioscience and pharmacology can negatively impact nurses in extended prescribing roles, and novice prescribers must improve their knowledge and skills in risk assessment and medication management to develop expertise in decision-making (Lim et al., 2018).

Although antimicrobial guidelines were considered important, NPs in the current study strongly favored an individualized approach, selecting a regimen based on efficacy, dosing frequency, route of administration, and appropriateness for the patient. Cognitive difficulty occurred when NPs balanced the risks versus benefits of an antibiotic regimen with these patient factors, prioritizing the patient's schedule and adherence over guideline recommendations. Antimicrobial stewardship has not been shown to greatly influence NP's decision to prescribe antibiotics (Hannigan et al., 2012; Wright et al., 2019) with efficacy, tolerability (Hannigan et al., 2012; Goolsby, 2007), and adherence (Hannigan et al., 2012; Wright et al., 2019) a higher priority when selecting antibiotic treatment. This is supported by findings that suggest NPs favor a holistic approach, taking time in their consultations with patients to gain an understanding of their health requirements (Offredy, 1998). These aspects build trust between the nurse and patient and enable shared-decision making, increasing the likelihood of a positive health outcome, but may also result in patients being prescribed an antibiotic that is not first-line.

A significant cognitive difficulty for NPs in our study was dealing with patient demand for antibiotics. This was more evident in less experienced NPs who expressed poorer prescribing confidence. Where patients persisted, NPs made use of delayed prescribing. Patient demand is often a significant driver for the unnecessary prescribing of antibiotics (Dempsey et al., 2014; Cabral et al., 2016), and delayed prescribing can be a safe and effective way to manage this. However, a clear rationale and prescribing confidence are required to implement this approach, as prescribing antibiotics ‘just in case’ is associated with increased antimicrobial resistance (Courtenay et al., 2019; Dexter and Mortimore, 2020). Inconsistent prescribing between professions is a significant barrier when attempting to negotiate a treatment plan with patients (Rowbotham et al., 2012; Williams et al., 2018), and NPs often reflect the prescribing practices of GPs, who “soften” to antibiotic demand over time, neglecting to provide patients education on antibiotic use due to workload pressure (Hannigan et al., 2012). NPs, however, have reported not being influenced by the same workload pressures as GPs, and hence were not influenced by patient pressure when prescribing (Rowbotham et al., 2012). Strategies to curb antibiotic demands typically include requesting support from colleagues to reinforce their no-prescribing decision (Rowbotham et al., 2012).

## 5 Limitations

This study has several limitations. The ACTA framework is a time-consuming process for both the researcher and the participant. Although the method recommends that three to five experts are sufficient to solicit rich data due to the depth of the interview and analysis, it cannot be representative of all NPs. The inclusion criteria were restricted to NPs who had completed the enhanced NP curriculum that incorporated antimicrobial stewardship and those who routinely prescribed antibiotics. Therefore, the prescribing practices of earlier registered NPs and NPs practicing in niche specialties are not reflected in this study. The study was also vulnerable to self-reported bias. Data gathered were subjective to the perspective of the participants and NPs were able to selectively reveal or suppress information from the researchers. Additionally, the ACTA method relies on memory recall of a challenging situation, and verbal reports may not fully represent a cognitively demanding situation or experience. While the simulation interview, which is part of the ACTA toolkit, was not essential for this study, simulating the process of antibiotic prescribing by NPs may have generated further data that participants may subconsciously neglect through simple recall. Student researchers may also have introduced their own biases or assumptions when analyzing the data.

## 6 Conclusion

The ACTA framework provided a basis for an examination of the cognitive processes of NPs when prescribing antibiotics and has given valuable insight into the cognitive strategies NPs in New Zealand use to meet those demands. The study identified areas where continued professional development can be targeted to support antibiotic prescribing by NPs, showing that they would likely benefit from resources that are freely available, and which reflect national or local data on safe and effective antimicrobial use. To overcome the cognitive demands of antimicrobial prescribing, and minimize the risk of antimicrobial resistance, NPs must have access to guidelines and protocols that address treatment for patients with complex medical and social histories. The application of targeted education resources and clinical pathways to help diagnostic uncertainty for NPs remains unclear. Therefore, further studies are needed to explore the impact of educational interventions on NPs’ future prescribing practice.

## 7 Implications for practice

The findings of this study highlight factors that are cognitively demanding for NPs when making antimicrobial prescribing decisions. Although NPs are responsible for developing their skill and knowledge competence to provide safe and effective prescribing, educators play an important role in building prescriber confidence. Simply creating more awareness of antimicrobial stewardship does not extend far enough when NPs are faced with complex patient populations. Educators, therefore, need to be attuned to where the specific learner needs are and how best to target learning opportunities, particularly in high-risk population groups. This study has confirmed the need for further pharmacology and therapeutics knowledge upskilling, as well as the development of practical skills, to better enable NPs to balance the risks and benefits of antimicrobial prescribing. This is particularly important where guidelines are not readily available and where NPs may engage in either/or thinking, limiting their choices by what they know, rather than exploring what other options may be more suitable. Creating more awareness around the appropriateness of delayed prescribing is crucial to address a knee-jerk reaction of ‘just in case’ prescribing, and ensure NPs are prescribing confidently intending to reduce antimicrobial resistance. To integrate these factors into the NP curricula requires a solid understanding of where the gaps in antimicrobial stewardship exist; however, few studies have considered NP education in NZ, and little is currently known how best to support NPs in this learning.

## 8 Further research

Accessibility to reputable local antimicrobial resources that address treatment for complex patients could help bolster prescribing confidence. Further research could help to identify where resource gaps are and how NPs want to engage with guidelines and protocols. Opportunities to explore cognitive demands in NPs with more than 2 years of experience would be helpful to identify whether antimicrobial prescribing demands are similar at a more advanced level. Research may suggest how expert nurses build resilience to patient demand and how antibiotic prescribing decision-making develops with experience.

## Funding

No external funding.

## Declaration of Competing Interest

None.

## Appendix Supplementary materials

Image, application 1
