# Supplementary material for: Assessing antibiotic prescribing in nurse practitioners: Applied cognitive task analysis
Source: Int J Nurs Stud Adv. 2022 Sep 25;4:100101. doi: 10.1016/j.ijnsa.2022.100101 (PMC11080434; doi:10.1016/j.ijnsa.2022.100101)
Supplement: Supplementary file 1 [file mmc1.docx]

Table 2: Master knowledge audit table of the cognitive demands of antibiotic prescribing

|  | **Aspects of Expertise** | **Cues and Strategies** | **Why Difficult** |
| --- | --- | --- | --- |
| **Big Picture** | Applying clinical and non-clinical knowledge to determine whether to treat with antibiotics.  Considering factors that guide treatment e.g.   - Patient demographics - Social circumstances - Adherence to treatment plan - Risk factors for special populations. | Clinical assessment of the infection:   - Severity and location - Strain of causative bacteria - Risks vs benefits of antibiotics - Formulation - Course duration   Pertinent considerations through patient consultation and clinic records.   - Medical/medication history - Contraindications - Social situation e.g. socioeconomic status - Health literacy and adherence   Use of guidelines or advice from colleagues in deciding treatment or dosing regimen. | Patients' circumstances influence decision-making:   - Special population group e.g. children - Comorbidity - Health literacy - Social situations - Non-adherence / reluctance   Risk of complaints when expectations are not met, or initial plan does not work.  Pressure from superior to prescribe.  NPs feel more accountable when prescribing.  Weighing up risks and benefits of not prescribing antibiotics. |
| **Noticing** | Recognising severity, and risk of infections in certain populations and changes in clinical status.  Integrating patient examination with unusual clinical presentation. | Clinical observation of the patient. Interpretation of lab values or diagnostic tests (X-ray, blood test). Pattern recognition.  Combining patient history with physical examination to formulate a treatment plan based on infection severity.  Improve patient health literacy. | Some conditions require in-depth clinical assessment or investigations to make a diagnosis.  Pattern recognition not always accurate. |
| **Job Smarts** | Working efficiently by focusing on relevant clinical information and/or tests.  Building a strong rapport with patients, and managing patient pressure to prescribe antibiotics through education.  Working in a multidisciplinary team to optimise patient outcomes. | Effective use of time by identifying typical signs of infection through pattern recognition.  Patient education to increase health literacy.  Referring to other clinicians or to their primary care practitioner. | Novices order unnecessary tests.    Balancing time with patients and other commitments in the practice.  Inadequate patient education on management of their condition can result in patient readmittance, creating work for other clinicians.  Patients use consultations to insist on antibiotics. |
| **Opportunity/ Improvising** | Having confidence in knowledge and available resources such as guidelines, colleagues and further assessments to make decisions and investigate conflicting information.  Taking opportunities positively to extend scope of practice and clinical knowledge, but being aware of limitations and knowing when to seek help. | Seek advice from or referral to other clinicians e.g. pharmacists, consultants, and the infectious disease team.  Keep abreast of knowledge through self-study.  Supplying pre-packaged antibiotics to patients who may not be able to access a pharmacy to obtain their treatment.  Reassessing treatment e.g. extending antibiotic course if no improvement occurs despite patient’s adherence to treatment or changing to another antibiotic. | Guidelines do not always have comprehensive or specific coverage for complicated presentations or uncommon infections.  Doing self-study in own time.    Broadening scope means more responsibility.  Interpreting significance of drug-drug and drug-patient interactions. |
| **Past and Future** | Use of knowledge and experience of patient risk factors to guide a treatment plan.  Pattern recognition. | Clinical examination and interpretation of lab data. Past experience.  Advice from other prescribers to gather another perspective.  Including patient's goals of treatment and expectations when planning treatment. Back-pocket prescribing.  Practicing assertive and effective communication regarding decisions. | Differential diagnosis of similar conditions with the use of a more comprehensive history taking and patient assessment.  Balancing need for empiric antibiotics and antimicrobial stewardship.  Patient expectation/ demand can be influenced by prescribing practice and culture elsewhere. |
| **Self-monitoring** | Reflecting on practice and taking responsibility for self-directed learning.  Acknowledge personal limitations and know when to seek advice. | Continuing education/self-directed study.  Audit prescribing by checking decision against the cultures and whether changes were made after referral to specialist care. | National and international resources can be difficult to access and costly with limited support from the workplace for continuing education. |
| **Antimicrobial Stewardship** | Understanding the importance of antibiotic resistance.  Consequences of not practicing antimicrobial stewardship. | Prescribing only when there is an indication. Consider narrow or broad-spectrum antibiotics and bacteria sensitivity.  Follow up patients to monitor for clinical improvement. Consider non-adherence.  Prescribing back pocket.  Standing firm on clinical decisions and discussing options with consultants. | Balancing patient expectation and antimicrobial stewardship.  Non-adherence may be due to the challenge of following up the patient’s progress and monitoring their adherence.  Patients may not finish the antibiotic course due to poor of understanding of resistance.  Pressure from senior clinicians to prescribe. Clinicians that prescribe antibiotics for viral infections. |
| **Anomalies** | Considering patient demographics (culture, age), medical status and expectations to ensure safe prescribing and mutual treatment plan. | Patient education on importance of closely controlling antibiotic use. | Patients from different cultural background have different expectations around antibiotic treatment.  Communicating to patients with limited English and poor health literacy. |
